# EARLY INPATIENT REHABILITATION IN GERIATRIC PATIENTS: A SYSTEMATIC REVIEW OF OUTCOME MEASURES

**DOI:** 10.1093/geroni/igz038.604

**Published:** 2019-11-08

**Authors:** Klaus Hauer, Patrick Heldmann, Christian Werner

**Affiliations:** 1 Agaplesion Bethanien Hospital, Geriatric Center at the Heidelberg University, Heidelberg, Germany; 2 Network Aging Research (NAR), Heidelberg University, Heidelberg, Germany

## Abstract

Selecting appropriate outcome measures for multimorbid, acutely hospitalized geriatric patients poses specific challenges, which may have caused inconsistent findings of previous intervention trials on early inpatient rehabilitation. The objective of this review was to describe primary outcome measures used in randomized controlled trials (RCTs) on early rehabilitation in older hospital patients, to analyze their matching to intervention programs, and to evaluate the effects of matching on the main findings of these RCTs. A systematic literature search was conducted in PubMed, Cochrane CENTRAL, CINAHL, and PEDro databases. Inclusion criteria were: RCT, patients aged ≥ 65 years, admission to hospital, physical exercise intervention, and primary outcome measure during hospitalization. Two independent reviewers extracted the data, assessed the methodological quality, and analyzed the matching of primary outcome measures to the intervention, study sample, and setting. Main study findings were related to the results of the matching procedure. In 28 included articles, 33 different primary outcome measures were identified, which we grouped into six categories: functional status, mobility status, hospital outcomes, adverse clinical events, psychological status, and cognitive functioning. Outcome measures differed considerably within each category showing a large heterogeneity in their matching to the intervention, study sample, and setting. Outcome measures that specifically matched the intervention contents were more likely to document intervention-induced benefits. Mobility instruments seemed to be the most sensitive outcome measures to reveal such benefits. High specificity (optimized match) of outcome measures and intervention contents is a key factor to reveal benefits of early rehabilitation in acutely hospitalized geriatric patients.

